# Regional contrast agent quantification in a mouse model of myocardial infarction using 3D cardiac T_1 _mapping

**DOI:** 10.1186/1532-429X-13-56

**Published:** 2011-10-05

**Authors:** Bram F Coolen, Tessa Geelen, Leonie EM Paulis, Klaas Nicolay, Gustav J Strijkers

**Affiliations:** 1 Biomedical NMR, Department of Biomedical Engineering, Eindhoven University of Technology, PO BOX 513, 5600MB, Eindhoven, the Netherlands

## Abstract

**Background:**

Quantitative relaxation time measurements by cardiovascular magnetic resonance (CMR) are of paramount importance in contrast-enhanced studies of experimental myocardial infarction. First, compared to qualitative measurements based on signal intensity changes, they are less sensitive to specific parameter choices, thereby allowing for better comparison between different studies or during longitudinal studies. Secondly, T_1 _measurements may allow for quantification of local contrast agent concentrations. In this study, a recently developed 3D T_1 _mapping technique was applied in a mouse model of myocardial infarction to measure differences in myocardial T_1 _before and after injection of a liposomal contrast agent. This was then used to assess the concentration of accumulated contrast agent.

**Materials and methods:**

Myocardial ischemia/reperfusion injury was induced in 8 mice by transient ligation of the LAD coronary artery. Baseline quantitative T_1 _maps were made at day 1 after surgery, followed by injection of a Gd-based liposomal contrast agent. Five mice served as control group, which followed the same protocol without initial surgery. Twenty-four hours post-injection, a second T_1 _measurement was performed. Local ΔR_1 _values were compared with regional wall thickening determined by functional cine CMR and correlated to *ex vivo *Gd concentrations determined by ICP-MS.

**Results:**

Compared to control values, pre-contrast T_1 _of infarcted myocardium was slightly elevated, whereas T_1 _of remote myocardium did not significantly differ. Twenty-four hours post-contrast injection, high ΔR_1 _values were found in regions with low wall thickening values. However, compared to remote tissue (wall thickening > 45%), ΔR_1 _was only significantly higher in severe infarcted tissue (wall thickening < 15%). A substantial correlation (*r *= 0.81) was found between CMR-based ΔR_1 _values and Gd concentrations from *ex vivo *ICP-MS measurements. Furthermore, regression analysis revealed that the effective relaxivity of the liposomal contrast agent was only about half the value determined *in vitro*.

**Conclusions:**

3D cardiac T_1 _mapping by CMR can be used to monitor the accumulation of contrast agents in contrast-enhanced studies of murine myocardial infarction. The contrast agent relaxivity was decreased under *in vivo *conditions compared to *in vitro *measurements, which needs consideration when quantifying local contrast agent concentrations.

## Background

Mouse models of myocardial infarction are widely used to investigate the cascade of events occurring after myocardial ischemia/reperfusion injury. Cardiovascular magnetic resonance (CMR) has proven to be the modality of choice for such studies, as it can produce high-resolution information on both global and local indices of cardiac pathology [1-3]. Also, MRI contrast agents are increasingly applied as they allow monitoring of various kinds of disease-related processes on cellular and even molecular level, such as cell death, inflammation and fibrosis [4-6].

For commonly used T_1_-weighted imaging, the accumulation of paramagnetic contrast agent is associated with local changes in signal intensity. However, signal intensity is an indirect and qualitative measure of concentration, because it is influenced by several other factors, such as coil sensitivities, gain calibration, sequence timing parameters and fluctuations in ECG- or respiratory triggering intervals. Consequently, there is a growing interest for the application of T_1 _mapping protocols in contrast-enhanced studies of myocardial infarction using T_1 _contrast agents. Bohl et al. [7] used a Look-Locker T_1 _mapping method to rapidly measure changes in T_1 _in both remote and infarct areas after injection of Gd-DTPA. Quantitative knowledge of T_1 _was translated to the inversion time that resulted in optimal contrast in late Gd enhancement (LGE) imaging. Similar implementations of this method have also been used to dynamically measure T_1 _after infusion of Mn^2+^, an ion that acts as a Ca^2+ ^analogue, to report on Ca^2+ ^handling [8,9].

In this study, we investigated whether quantitative T_1 _measurements can also serve for the assessment of local contrast agent concentrations. This is particularly valuable for molecular imaging, where one aims to relate local amounts of contrast agent to the presence of certain disease markers. We recently presented a novel imaging protocol for measuring mouse myocardial T_1 _with whole-heart coverage, based on retrospective triggering and variable flip angle analysis [10]. Good reproducibility of measuring regional T_1 _was found between measurements at successive days; however, limited data was available concerning changes in T_1 _in infarcted myocardium upon injection of a Gd-based contrast agent. Here, we hypothesize that this method provides an accurate read-out of regional contrast agent induced changes of myocardial T_1 _in a mouse model of ischemia/reperfusion injury and allows for determination of local contrast agent concentrations. To test this hypothesis, we measured the accumulation of a liposomal contrast agent after myocardial ischemia/perfusion injury and quantified local changes in T_1 _using three-dimensional T_1 _mapping. Then, ΔR_1 _values derived from pre- and post-contrast *in vivo *T_1 _maps were correlated to contrast agent concentrations obtained from *ex vivo *inductively coupled plasma mass spectrometry (ICP-MS).

The use of a liposomal contrast agent allowed for T_1 _measurements several hours after contrast agent injection at a time point at which the contrast agent was almost cleared from the blood circulation and did not contaminate the measurement of myocardial T_1_. In this application, myocardial contrast agent concentrations were stable for an extended period of time, which increased the available imaging time and allowed for full 3D coverage of the heart. Moreover, the use of liposomes in this experimental mouse model of myocardial infarction provided the opportunity to perform relevant validation experiments concerning *in vivo *quantification of the actual contrast agent concentration and relaxivity properties in the heart.

## Materials and methods

### Contrast agent preparation and characterization

#### Preparation

Paramagnetic liposomes were prepared as described earlier [11]. In short, liposomes were prepared by lipid film hydration of a lipid mixture containing 100 μmol of total lipid. This mixture consisted of gadolinium-diethylenetriaminepentaaceticacid-bis(stearylamide) (Gd-DTPA-BSA, Gateway Chemical Technology, St. Louis, MO, USA), 1,2-distearoyl-*sn*-glycero-3-phosphocholine (DSPC, Lipoid GmbH, Ludwigshafen, Germany), cholesterol (Avanti Polar Lipids, Alabaster, AL, USA), 1,2-distearoyl-*sn*-glycero-3-phospoethanolamine-*N*-[methoxy(poly(ethylene glycol))-2000] (PEG2000-DSPE, Lipoid GmbH) and 1,2-distearoyl-*sn*-glycero-3-phospoethanolamine-*N*-[maleimide(poly (ethylene glycol))-2000] (Mal-PEG2000-DSPE, Avanti Polar Lipids) at a molar ratio of 0.75/1.1/1/0.075/0.075. The resulting vesicles were sized by extrusion through 200 nm filters (four times), followed by extrusion through two 100 nm filters (ten times). Liposomes were concentrated by centrifugation at 55000 rpm and 4°C for 1 hour. Finally, liposomes were resuspended in HEPES-buffered saline (20 mM HEPES and 135 mM NaCl, pH 7.4) at a concentration of approximately 70 mM of total lipid.

#### Characterization

The final total lipid concentration was determined with a phosphate analysis according to Rouser [12]. Dynamic light scattering (DLS) was performed at 23°C with a Malvern ZetaSizer Nano S and the resulting hydrodynamic diameter of the liposomes was approximately 115 nm. Longitudinal relaxivity r_1 _at 9.4T was measured in HEPES-buffered saline at 37°C. For this purpose, T_1 _measurements were performed on samples with various concentrations of Gd-DTPA-BSA (0.01-1.25 mM) using a snapshot-FLASH T_1 _mapping method [13]. The following parameters were used: scan repetition time = 15 s, TR/TE = 4.0/2.0 ms, α = 15°, Nsegments = 32, slice thickness = 1 mm. A total of 60 inversion times were sampled ranging from 72 to 4792 ms.

### *In vivo *measurements

Mice were obtained from a commercial breeder (Charles River, Paris, France) and housed under controlled conditions. A total of 13 Swiss mice (male, 33 ± 2 g) were used in this study. In the infarct group (N = 8), mice underwent ischemia/reperfusion surgery by a 30-minute ligation of the left anterior descending (LAD) coronary artery. One day after surgery, CMR was performed, consisting of 3D T_1 _mapping measurements to assess baseline T_1_, as well as 3D CINE measurements for determination of wall- thickening. This was followed immediately by injection of a liposomal contrast agent through the tail vein (0.05 mmol Gd/kg). Five control mice served as control group, in which the same CMR measurements and contrast agent administration were performed without previous surgery. Twenty-four hours post-injection, a second T_1 _measurement was performed using the same protocols. The mice were then sacrificed and the heart was excised to measure the Gd content ex vivo. The study protocols were approved by the local animal ethical committee.

CMR was performed with a 9.4 T Bruker experimental scanner using a 72-mm-diameter volume transmit coil in combination with a mouse cardiac phased-array surface coil (Bruker BioSpin GmbH, Ettlingen, Germany). The latter consists of a 2 by 2 array of square coil elements, each sized 16 × 16 mm^2^. 3D T_1 _mapping was performed as published previously [10] using retrospective triggering (3D IntraGate) with variable flip angles. In the current study, three different flip angles α were used (α = 2°, 8° and 14°). This was shown to produce highly reproducible myocardial T_1 _maps, in which the 95% confidence interval for ΔR_1 _at baseline values was only 0.15 s^-1^. After pre-contrast T_1 _measurements, the same sequence was performed in black-blood mode and α = 8° for assessing wall thickening values. For this particular purpose, black-blood was chosen over bright-blood scans, because of superior wall delineation. On the other hand, black-blood scans are less suitable for T_1 _mapping (see Discussion). Although the use of retrospective triggering enables reconstruction of an arbitrary number of cardiac frames, a sufficient number averages is needed for each frame. With an imaging time of 20 minutes, reconstruction of 12 cardiac frames provided good quality black-blood 3D CINE images for determining wall thickening values from end-diastolic and end-systolic frames. The total imaging time for each animal was approximately 90 minutes.

### Data analysis

Pre- and post-contrast myocardial 3D T_1 _maps were segmented according to the AHA 17-segment model [14] using a custom-built segmentation algorithm (Mathematica 7.0, Wolfram Research, Champaign, IL, USA). Segmentation was based on the high flip angle T_1_-weighted scan, in which blood-myocardium contrast was highest. For each segment, mean pre- and post-contrast R_1 _values were calculated (R_1 _= 1/T_1_), as well as corresponding ΔR_1 _values (R_1_^post ^- R_1_^pre^). Additionally, wall thickness (WT) of all segments was determined from the black-blood 3D IntraGate scans in both end-diastolic (ED) and end-systolic (ES) cardiac phases. Systolic wall thickening (SWT) values were defined as (WT^ES^/WT^ED ^- 1) * 100%.

### *Ex vivo *Gd quantification

After post-contrast CMR measurements, mice were sacrificed through cervical dislocation. From all mice in the infarct group, the heart was excised and stored in the freezer at -80°C until further use. A mid ventricular slice containing the infarct area (2.5 mm above the apex) was then cut from the heart using a mouse heart slicer (Zivic Instruments, Pittsburgh, PA, USA). The slice was subsequently cut into four segments (septal, posterior, lateral and anterior), each of which was weighed and put in a vial. For all segments, the Gd content (in nanograms) was determined with ICP-MS. The Gd concentration ([Gd]) was then calculated in each segment using the specific gravity of myocardial tissue (1.05 g/ml) and the molar weight of Gd (157.25 g/mol).

### Statistics

Statistical analysis was done with SPSS 17.0 (SPPS, Inc., Chicago, IL, USA) and JMP 9.0 (SAS Institute, Cary, NC, USA). All values are reported as mean ± standard deviation. All tests were performed on the R_1 _values, rather than on T_1 _values. The level of statistical significance was set at p = 0.05 for all tests. Pre- and post-contrast R_1_, as well as ΔR_1 _values were considered separately. For these parameters, significant differences between infarct, remote and control areas were tested with a one-way ANOVA and *post-hoc *Tukey test. Note that infarct segments were defined as those segments with a corresponding wall thickening value smaller than 15%, whereas for remote regions, the lower threshold was set at 45%. Differences in ΔR_1 _as function of wall thickening were also tested using a one-way ANOVA and *post-hoc *Tukey test. To correlate regional ΔR_1 _values as estimated from *in vivo *CMR data with the Gd concentrations as measured by *ex vivo *ICP-MS, orthogonal regression was performed. This was preferred over linear regression, because errors in both measurement techniques should be taken into account.

## Results

**Figure 1:**
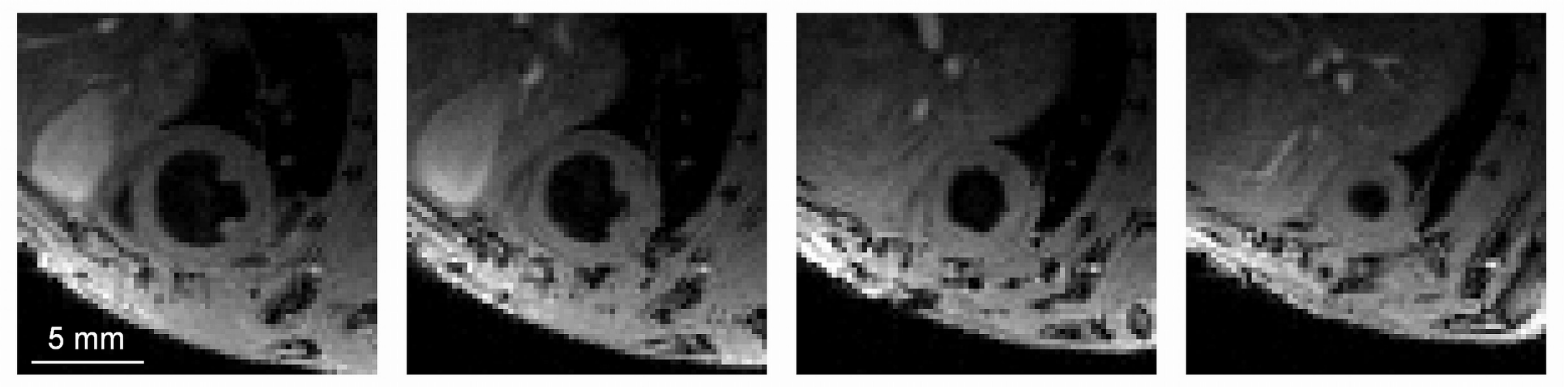
**3D black-blood retrospectively triggered CINE images used for determination of wall thickening**. 3D black-blood retrospectively triggered CINE images used for determination of wall thickening. Images from left to right represent different slices through the heart towards the apex (movie provided in Additional File 1).

Figure 1 shows end-diastolic black-blood images of a representative mouse of the infarct group in four different slices reconstructed from the 3D imaging volume. The entire CINE series containing all cardiac frames is provided as a supplemental movie in Additional File 1. Blood suppression was excellent throughout the whole 3D volume, resulting in good delineation of the cardiac wall without presence of blood flow artifacts. Typically, akinetic regions were found in the postero- and anterolateral wall.

**Figure 2:**
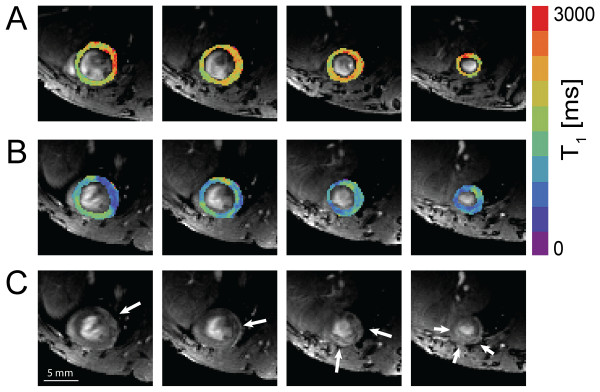
**Pre-contrast myocardial T**_**1 **_**(A), post-contrast myocardial T**_**1 **_**(B), and post-contrast T**_**1**_**-weighted images (C) in a mouse with myocardial infarction**. Pre-contrast myocardial T_1 _(A), post-contrast myocardial T_1 _(B), and post-contrast T_1_-weighted images (C) in a mouse with myocardial infarction. Arrows mark infarct regions showing contrast enhancement in post-contrast T_1_-weighted imaging. Images represent the same slices as in Figure 1.

Representative maps from pre- and post-contrast myocardial T_1_, as well as corresponding post-contrast T_1_-weighted images are shown in Figure 2. Akinetic regions apparent from Figure 1 can already be distinguished in the pre-contrast T_1 _maps (Figure 2A) from an increase in T_1_. However, they are more evident in the post-contrast images from a large increase in signal intensity (Figure 2C, arrows) and corresponding significant decrease in T_1 _(Figure 2B).

**Figure 3:**
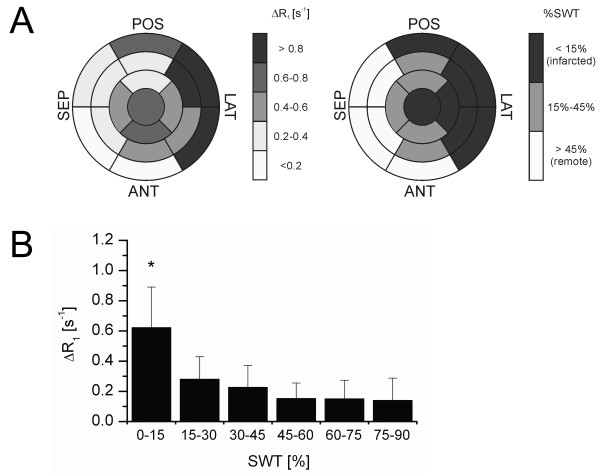
**Relation between wall thickening and contrast agent accumulation.** (A) Bull's eye plots of ΔR_1 _and wall thickening (SWT) values corresponding to the animal in Figure 1. Regions with SWT < 15% are considered infarcted, whereas remote regions have SWT > 45%. (B) Relation between wall thickening and their corresponding ΔR_1 _values (mean value of all animals, * p < 0.01).

Figure 3A shows Bull's eye plots of regional values of ΔR_1 _(R_1_^post ^- R_1_^pre^) and wall thickening of the same mouse as in Figure 2. The latter was divided into three categories, where SWT values < 15% and > 45% were considered 'infarcted' or 'remote', respectively [1,15]. Regions of low SWT matched with regions of high ΔR_1 _values, induced by the presence of the paramagnetic liposomes. The mean ΔR_1 _as function of SWT is plotted in Figure 3B. ΔR_1 _decreased rapidly with increasing percentage of wall thickening, but only severely infarcted regions revealed significantly elevated ΔR_1 _values compared to remote regions. For comparison, SWT in healthy control mice was 65.0 ± 12.3%.

**Figure 4:**
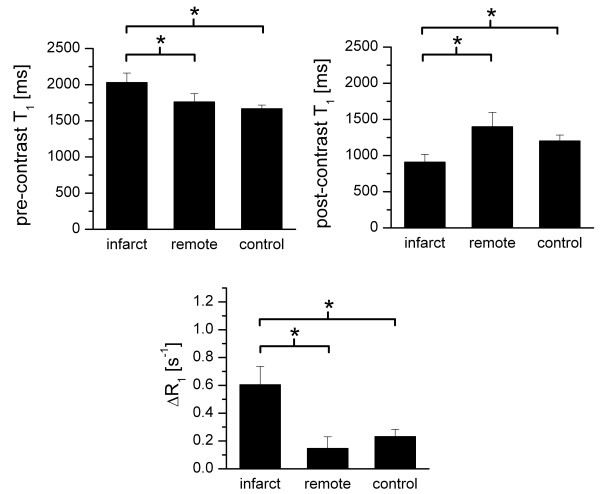
**Statistical analysis**. Statistical analysis of pre-contrast T_1_, post-contrast T_1 _and ΔR_1 _for infarcted, remote and control tissue (* p < 0.01).

Statistical differences in pre-contrast T_1_, post-contrast T_1 _and ΔR_1 _between infarct, remote and control regions are elaborated in Figure 4. For all parameters, there was a significant difference (p < 0.01) between the infarct regions on the one hand and both control and remote regions on the other hand. In pre-contrast T_1 _scans, the mean T_1 _of infarcted myocardium was elevated (2030 ± 131 ms) as compared to remote (1761 ± 114 ms) and control values (1667 ± 50 ms). Post-contrast T_1 _values were decreased compared to pre-contrast T_1 _in infarct (910 ± 107 ms), remote (1400 ± 196 ms) as well as control (1203 ± 86 ms) regions. T_1 _was significantly lowest in the infarcted myocardium. The post- and pre-contrast longitudinal relaxation rate difference (ΔR_1_) was largest for the infarct region (0.60 ± 0.13 s^-1^). However, also in remote tissue and in control animals the contrast agent resulted in a significant ΔR_1 _(0.15 ± 0.08 s^-1 ^and 0.23 ± 0.05 s^-1^, respectively, not significantly different). Since the liposomal contrast agent does not extravasate from healthy blood vessels, these R_1 _changes in remote and control tissues are likely caused by contrast agent still circulating in the blood 24 hours post injection.

**Figure 5:**
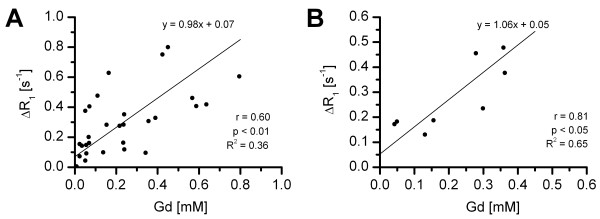
**Correlation between regional ΔR**_**1 **_**and Gd concentration.** Correlation between regional ΔR_1 _values determined by *in vivo *CMR and Gd concentration determined by *ex vivo *ICP-MS. The analysis was done either on all individual heart segments (A) or after averaging all values in each heart slice (B).

Figure 5 shows the relation between ΔR_1 _values determined by *in vivo *CMR and Gd concentrations determined by *ex vivo *ICP-MS. When considering all segments that were analyzed separately (Figure 5A), the Pearson correlation coefficient r was 0.6 with R^2 ^= 0.36. However, a more substantial correlation was found after averaging values for each heart slice (r = 0.81) with a corresponding R^2 ^of 0.65 (Figure 5B). Furthermore, a small but significant offset for ΔR_1 _was found in both analyses. The slope is the effective *in vivo *relaxivity of the liposomal contrast agent r_1 _= 1.06 mM^-1^s^-1^. This was significantly lower than r_1 _= 2.2 mM^-1^s^-1 ^(R^2 ^= 0.99) for the liposomes in suspension *in vitro*.

## Discussion

In this paper we described the use of 3D T_1 _mapping to assess myocardial relaxation rates after ischemia/reperfusion injury before and after administration of a liposomal contrast agent. This type of contrast agent is especially known for its high relaxivity per particle and long circulation half-life [16], but also for its prolonged retention in the infarcted tissue, allowing visualization of the contrast agent many hours after administration [17-19].

In this study, we chose to perform post-contrast measurements 24 hours after contrast agent injection. The 24 hours time period allowed for sufficient accumulation of contrast agent in the infarct area, while at the same time providing sufficient clearance of the contrast agent from the blood. Similar ΔR_1 _values in remote and control tissue however suggested that a small amount of contrast agent was still present in the blood. At later time points, full blood clearance would have been achieved, but probably at the expense of efflux of contrast agent from the infarction. Also, it is known that infarct development and healing is a very dynamic process, whereas pre- and post-contrast measurements are aimed at representing the same infarct status. Therefore we limited our T_1 _quantification to the 24 hours time period. Previous experiments have shown a negligible mean effect of repeated measurements on R_1 _[10]. In future experiments, pre- and post-contrast blood T_1 _measurements could be incorporated to better quantify the influence of blood contrast agent concentration levels on myocardial T_1 _changes.

The black-blood protocol used in this study for functional measurements could also have been used for myocardial T_1 _mapping. However, although black-blood imaging was very effective in suppressing signal from the blood (see Figure 1), it does not prevent exchange of blood magnetization with the myocardium and therefore does not eliminate contamination of myocardial signal due to presence of contrast agent in the blood. Also, we have previously shown that saturation of the blood results in dramatically lower effective myocardial T_1 _values [10].

Based on regional ΔR_1 _values, a significantly higher uptake of contrast agent was found in infarct regions compared to remote and control regions. The identification of regions being either 'infarcted' or 'remote' was based on wall thickening values determined from functional CINE measurements (Figure 1). We chose rather conservative threshold values for wall thickening (< 15% and > 45%) to ensure proper identification of infarct and remote areas [1,15]. Additionally, it was shown that the infarct area had a significantly higher T_1 _value already in pre-contrast scans. Previously, elevated T_1 _values in infarcted myocardium have been attributed to several different mechanisms. Some studies argue that formation of myocardial edema early after ischemia results in T_1 _increase due to a larger fraction of free water [7,20]. The increased wall thickness in the infarcted myocardium visible in end-diastolic images (Figure 1) indeed supports this theory. Others [21] claim that this increase of myocardial T_1 _is more likely caused by a decrease in perfusion, based on iso-intense remote and infarcted tissue in proton density weighted images. The use of a surface coil in this study creates a signal intensity gradient in our cardiac images, which made it difficult to assess proton density and corroborate or negate the latter hypothesis.

An ongoing topic within in the field of contrast-enhanced MR imaging is the actual quantification of regional contrast agent accumulation. With respect to small animal cardiac imaging, this is one of few studies attempting to correlate contrast agent induced ΔR_1 _values derived from *in vivo *CMR measurements with contrast agent concentrations determined by *ex vivo *analysis [9]. When analyzing all segments (4 in each mouse) separately, only a weak correlation was found. However, a substantial correlation was found when concentration values of all four segments within the slice were averaged. We believe that weak correlation of individual segments is caused by inaccuracies in cutting the heart slices into exactly the same segments as those of the *in vivo *CMR. Because of the generally sharp demarcations of the infarct, small errors in circumferential division and sectioning of the left ventricle can therefore lead to substantial differences in Gd concentrations. We therefore believe that a significant part of the variation is caused by inaccuracies of the cutting procedure. Reproducibility of regional T_1 _quantification of healthy myocardium using the current CMR protocol was demonstrated previously [10]. In the present study, we found the same myocardial T_1 _values for the control group, proving reproducibility of the method across studies. *Ex vivo *determined Gd concentrations correlated fairly well with *in vivo *ΔR_1 _values. Taken together, we therefore conclude that regional quantification of contrast agent concentrations is feasible.

At this point, the mechanism of contrast agent accumulation is believed to be passive accumulation throughout the increased extracellular space by extravasation of the liposomes through leaky vasculature in the reperfused injured myocardium [22]. Regression analysis interestingly showed that the effective relaxivity of the liposomes *in vivo *was lower compared to that determined *in vitro*. Reduced relaxivity could be mediated by the cellular environment of the myocardium, partial volume effects or compartmentalization of the liposomes [23]. Compartmentalization may result from phagocytosis of liposomes by macrophages. Whether or not phagocytosis occurs also depends on the liposomal formulation [18,22]. Future studies could focus on combining regional quantification of contrast agent concentrations with more detailed histological analysis on the cellular level to reveal the exact mechanisms of uptake. Importantly, *in vivo *relaxivity values must be considered for correct estimation of Gd concentrations from ΔR_1 _values.

## Conclusions

In summary, the quantitative T_1 _mapping technique described in this study provides a reproducible tool to monitor the accumulation of contrast agents in contrast-enhanced studies of murine myocardial infarction. A particular field of application is that of targeted contrast agents, which are used for mapping the spatial distribution of specific biomarkers. The contrast agent relaxivity was found to be lower than that measured *in vitro*, which needs consideration when quantifying local contrast agent concentrations.

## List of abbreviations

ANOVA: analysis of variance; ICP-MS: inductive coupled plasma mass spectrometry; LAD: left anterior descending; WT: wall thickness; SWT: systolic wall thickening.

## Competing interests

The authors declare that they have no competing interests.

## Authors' contributions

BC: study design, data acquisition, image analysis, statistical analysis, manuscript drafting. TG: study design, contrast agent preparation, manuscript drafting. LP: study design, manuscript drafting. KN: study design, manuscript drafting. GS: study design, manuscript drafting and principal investigator. All authors read and approved the final manuscript.

## Supplementary Material

Additional file 1 **3D black-blood CINE**. 3D black-blood CINE movie (12 frames) for determination of regional wall thickening. Click here for file
