# Supplementary figures and images for: Regional contrast agent quantification in a mouse model of myocardial infarction using 3D cardiac T1 mapping
Source: J Cardiovasc Magn Reson. 2011 Oct 5;13(1):56. doi: 10.1186/1532-429X-13-56 (PMC3207957; doi:10.1186/1532-429X-13-56)

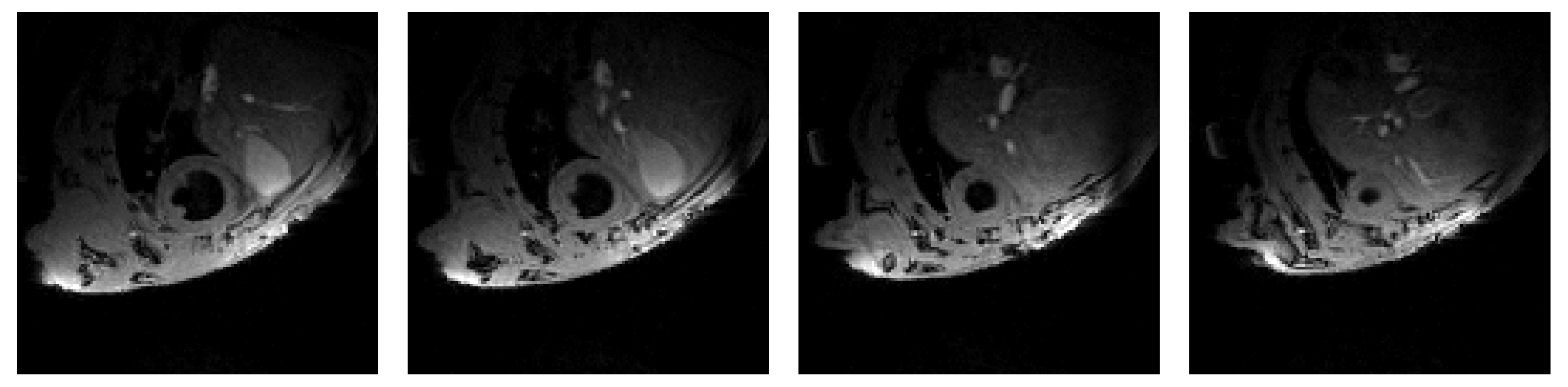

Supplement: Additional file 1 — 3D black-blood CINE. 3D black-blood CINE movie (12 frames) for determination of regional wall thickening. [file 1532-429X-13-56-S1.GIF]
